# Towards clinical grating-interferometry mammography

**DOI:** 10.1007/s00330-019-06362-x

**Published:** 2019-08-22

**Authors:** Carolina Arboleda, Zhentian Wang, Konstantins Jefimovs, Thomas Koehler, Udo Van Stevendaal, Norbert Kuhn, Bernd David, Sven Prevrhal, Kristina Lång, Serafino Forte, Rahel Antonia Kubik-Huch, Cornelia Leo, Gad Singer, Magda Marcon, Andreas Boss, Ewald Roessl, Marco Stampanoni

**Affiliations:** 1 ETH Zurich, Gloriastrasse 35, 8092 Zurich, Switzerland; 2 Paul Scherrer Institute, Forschungstrasse 111, 5232 Villigen, Switzerland; 3 Philips Research Hamburg, Röntgenstrasse 24-26, 22335 Hamburg, Germany; 4 HAW Hamburg, Ulmenliet 20, 21033 Hamburg, Germany; 5 Department of Radiology, Kantonsspital Baden, Im Ergel 1, 5404 Baden, Switzerland; 6 Interdisciplinary Breast Center, Kantonsspital Baden, Im Ergel 1, 5404 Baden, Switzerland; 7 Department of Pathology, Kantonsspital Baden, Im Ergel 1, 5404 Baden, Switzerland; 8 Institute for Diagnostic and Interventional Radiology, Universitätspital Zurich, Rämistrasse 100, 8091 Zurich, Switzerland

**Keywords:** Mammography, Phase contrast, Interferometry

## Abstract

**Objectives:**

Grating-interferometry-based mammography (GIM) might facilitate breast cancer detection, as several research works have demonstrated in a pre-clinical setting, since it is able to provide attenuation, differential phase contrast, and scattering images simultaneously. In order to translate this technique to the clinics, it has to be adapted to cover a large field-of-view within a clinically acceptable exposure time and radiation dose.

**Methods:**

We set up a grating interferometer that fits into a standard mammography system and fulfilled the aforementioned conditions. Here, we present the first mastectomy images acquired with this experimental device.

**Results and conclusion:**

Our system performs at a mean glandular dose of 1.6 mGy for a 5-cm-thick, 18%-dense breast, and a field-of-view of 26 × 21 cm2. It seems to be well-suited as basis for a clinical-environment device. Further, dark-field signals seem to support an improved lesion visualization. Evidently, the effective impact of such indications must be evaluated and quantified within the context of a proper reader study.

**Key Points:**

*• Grating-interferometry-based mammography (GIM) might facilitate breast cancer detection, since it is sensitive to refraction and scattering and thus provides additional tissue information.*

*• The most straightforward way to do grating-interferometry in the clinics is to modify a standard mammography device.*

*• In a first approximation, the doses given with this technique seem to be similar to those of conventional mammography.*

## Introduction

Mammography remains the mainstay imaging technique for screening and clinical evaluation of breast cancer [1] and the only modality that has demonstrated to decrease the number of deaths caused by breast cancer. However, mammography has limited sensitivity in dense breasts, due to the masking effect of overlapping tissue, and at the same time, women with dense breasts have a higher risk of breast cancer [2]. Notwithstanding, there is no consensus either on whether additional examinations should be carried out or on which in particular for women with dense breasts [3]. Potential additional or alternative methods include ultrasonography (US), magnetic resonance imaging (MRI), digital breast tomosynthesis (DBT), molecular breast imaging [4], and, maybe in the future, X-ray phase-contrast imaging (PCI). Both US and MRI can increase the sensitivity in dense breasts compared with mammography, but at the cost of reduced specificity [5, 6]. On the other hand, DBT has been shown to increase the performance in screening, but still has some limited sensitivity in extremely dense breasts, especially in the case of non-spiculated tumors [7], so PCI might hold potential as an alternative technique for mammography.

Unlike standard radiography, simply capable of measuring beam attenuation, PCI is sensitive to the refraction (i.e., change in direction or phase shift of the incoming X-ray beam) induced by the sample, providing a phase-contrast (PC) signal. In addition, grating-interferometry-based PCI (GI) is able to measure how much the sample scatters, yielding the so-called dark-field (DF) signal and delivering even more complementary information, which has shown to have potential for breast lesion detection [8]. In fact, in a reader study with 33 mastectomy samples, it was concluded that the GI images evidenced superior quality, with increased sharpness, lesion delineation, and microcalcification visibility [9]. However, it should be kept in mind that this study was performed at doses higher than the ones accepted in the clinics. Similarly, Anton et al [10] measured six mastectomy samples with GI and demonstrated that the DF images yielded higher contrast of important structures compared with attenuation.

These results have motivated the transfer of the GI technology to the clinics [11–13], which constitutes an engineering challenge. This task requires the adaptation of the method to cover a large field-of-view (FOV) within a limited exposure time, deliver a clinically acceptable dose [14], fulfill the ergonomic requirements, avoid increasing patient discomfort, and, most importantly, yield a higher diagnostic performance [15]. Taking all this into account, a straightforward strategy is to design a GI that fits into a standard mammography device [16]. In this work, we describe the technical properties of a novel clinically compatible GI-based mammography device based on an existing system and its first usage imaging mastectomy samples.

## Materials and methods

**Fig. 1:**
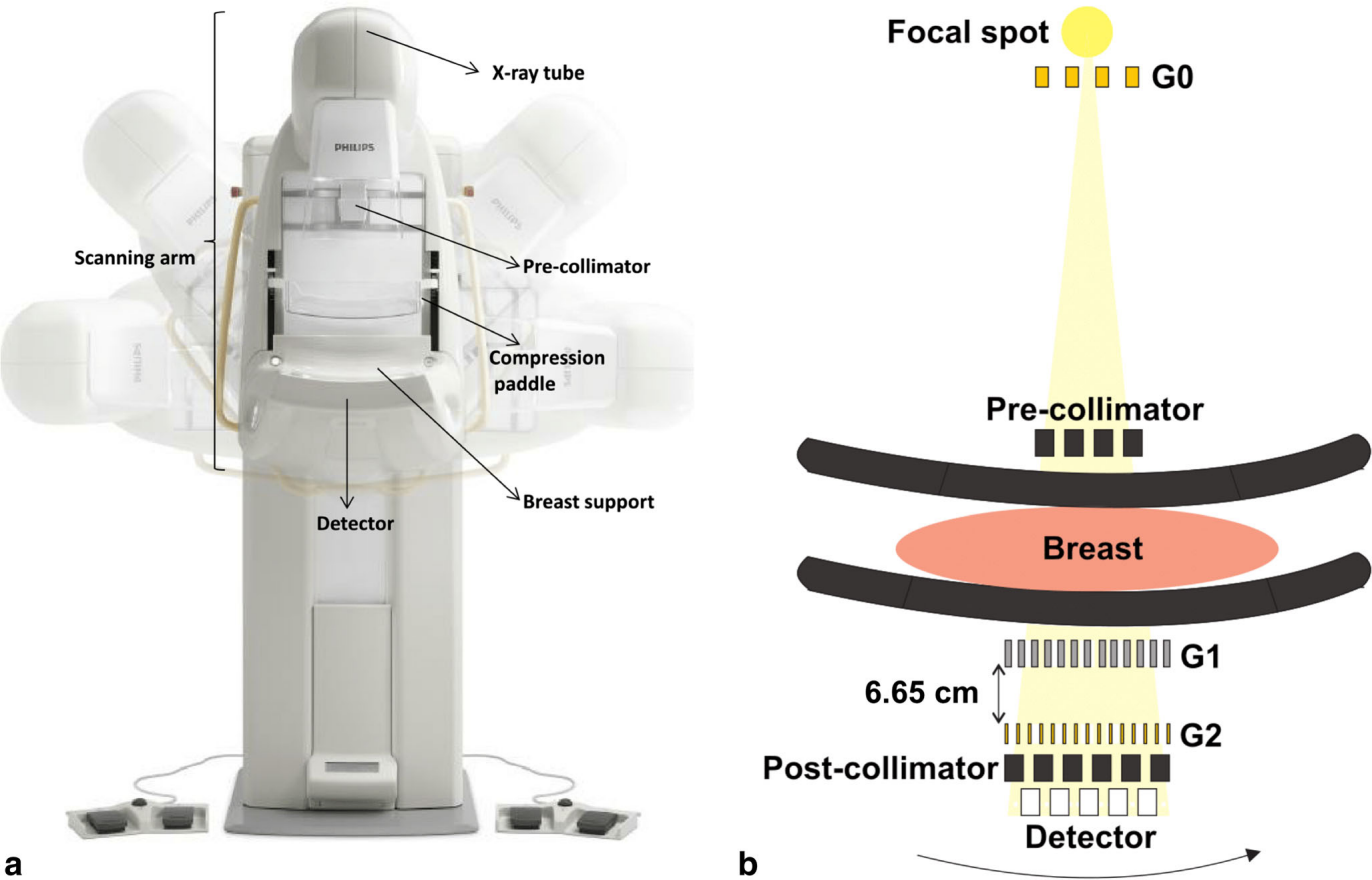
**a** Philips Microdose Mammography SI setup (reproduced from [17] and modified). The scanning arm moves to measure the breast, which is placed on the corresponding support. The setup allows the acquisition of all mammographic views. **b** Sketch of the GI installed on the Microdose setup

A device similar to a Microdose SI (Philips Diagnostic X-ray and Mammography Solutions) [15] was selected as the hosting device for the clinically compatible GI, mainly because of its slit-scanning geometry. The latter configuration allows using relatively small-area gratings (5 cm × 7 cm) to cover the required FOV (Fig. 1), which is rather large (26 × 21 cm^2^) to be spanned with a single grating, considering the corresponding engineering limitations [18]. The GI design parameters (Table 1) were optimized for the differential phase-contrast (DPC) signal following the procedure described in [17], but using a larger inter-grating distance to improve setup sensitivity, which could be achieved by modifying the pre-collimator height. The total setup length refers to the G0–G2 distance, whereas the design energy corresponds to the energy of the incoming spectrum the gratings were designed for to obtain the best achievable GI performance [17].

**Table 1:** GI prototype design parameters

Parameter	Value
GI length (cm)	55.6
G1-G2 distance (cm)	6.65
G0 period (*μ*m)	19.92
G1 period (*μ*m)	4.73
G2 period (*μ*m)	2.68
Design energy (keV)	26

**Fig. 2:**
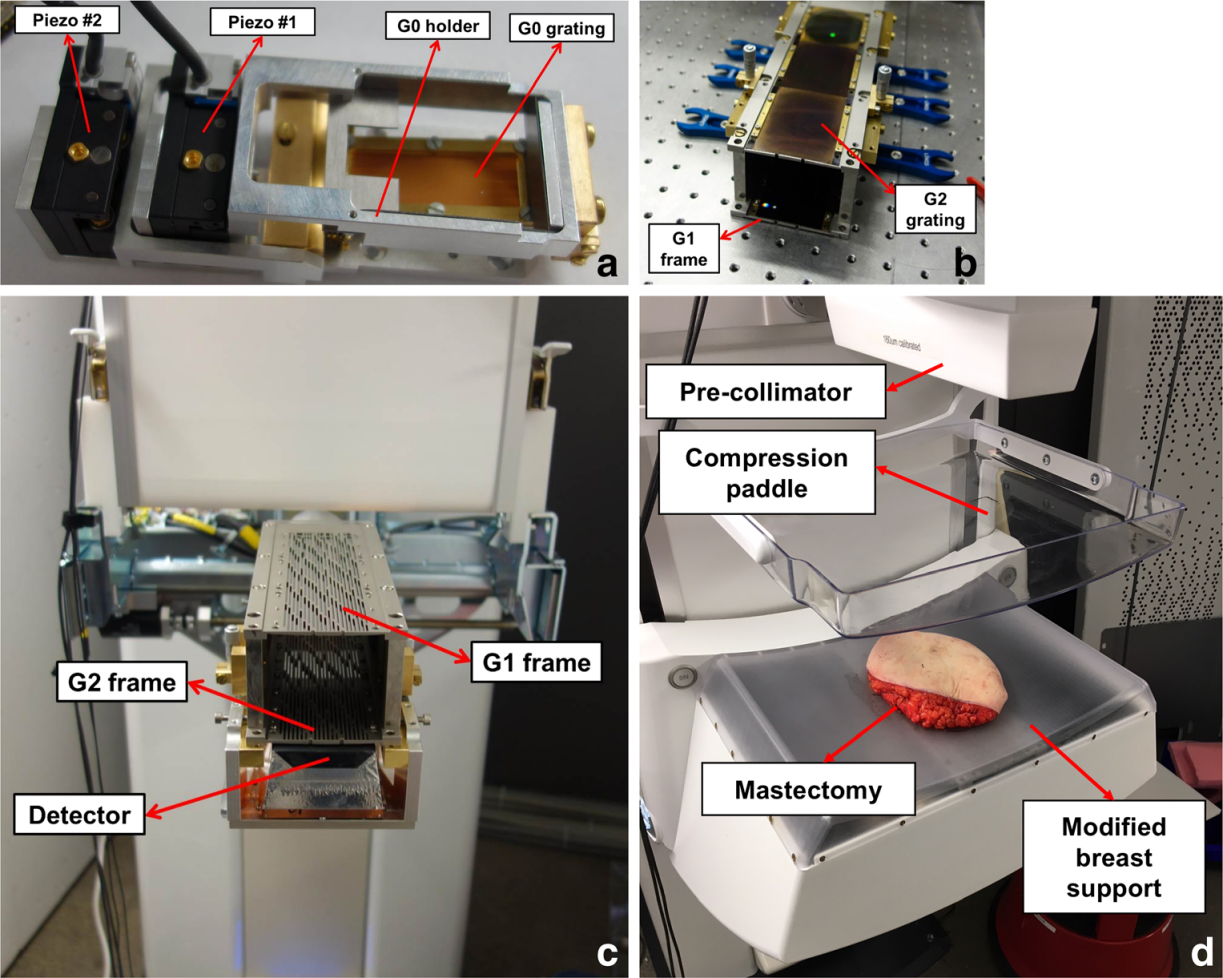
**a** G0 holder. **b** Fully assembled interferometer holder. **c** Interferometer holder mounted on the Microdose. In the latter, the gratings are facing downwards. **d** GI-retrofitted Microdose device. For the first time, a human mastectomy sample is measured on a fully clinically compatible GIM device

Gratings were mounted on a support that allowed for bending to compensate for the oblique incidence of the X-rays (patent number PCT/EP2018/062407) [19] and had a mask that matched the pre-collimator design so that bending could be supported while keeping the flux unaffected (Fig. 2c). For optimal grating alignment, vertical translation of the G0 support (Fig. 2a) was controlled by two piezoelectric motors, and the G1-G2 frames (Fig. 2b) were rotated using micrometer-precision screws. In addition, a dedicated biocompatible breast support that could accommodate the interferometer was commissioned (Fig. 2d).

The clinical study was approved by the responsible Ethical committee (EKNZ 2015’132) and written informed consent from the included patients was obtained. Four mastectomy specimens were imaged directly after surgical extraction at a tube voltage of 38 kVp with a total scanning time of 13.4 s in an uncompressed state. Although the latter is still above the typical measuring times for slit-scanning systems (5–8 s), it is still clinically compatible [14]. These exposure settings were selected in order to maximize the contrast-to-noise ratio on the PC and DF images, since due to their noise behavior, more incoming flux than in standard attenuation-based mammography is required to get the same signal quality [17].

Attenuation, DPC, and DF images were reconstructed with an iterative algorithm [16]. The attenuation and DPC signals were further fused [20] and processed with a contrast-boosting algorithm [21]. The latter was applied to the attenuation signal alone as well.

The air kerma and half-value layer were measured with a RaySafe device and used to calculate the mean glandular dose (MGD) for each sample according to the European Guidelines [14].

## Results

**Fig. 3:**
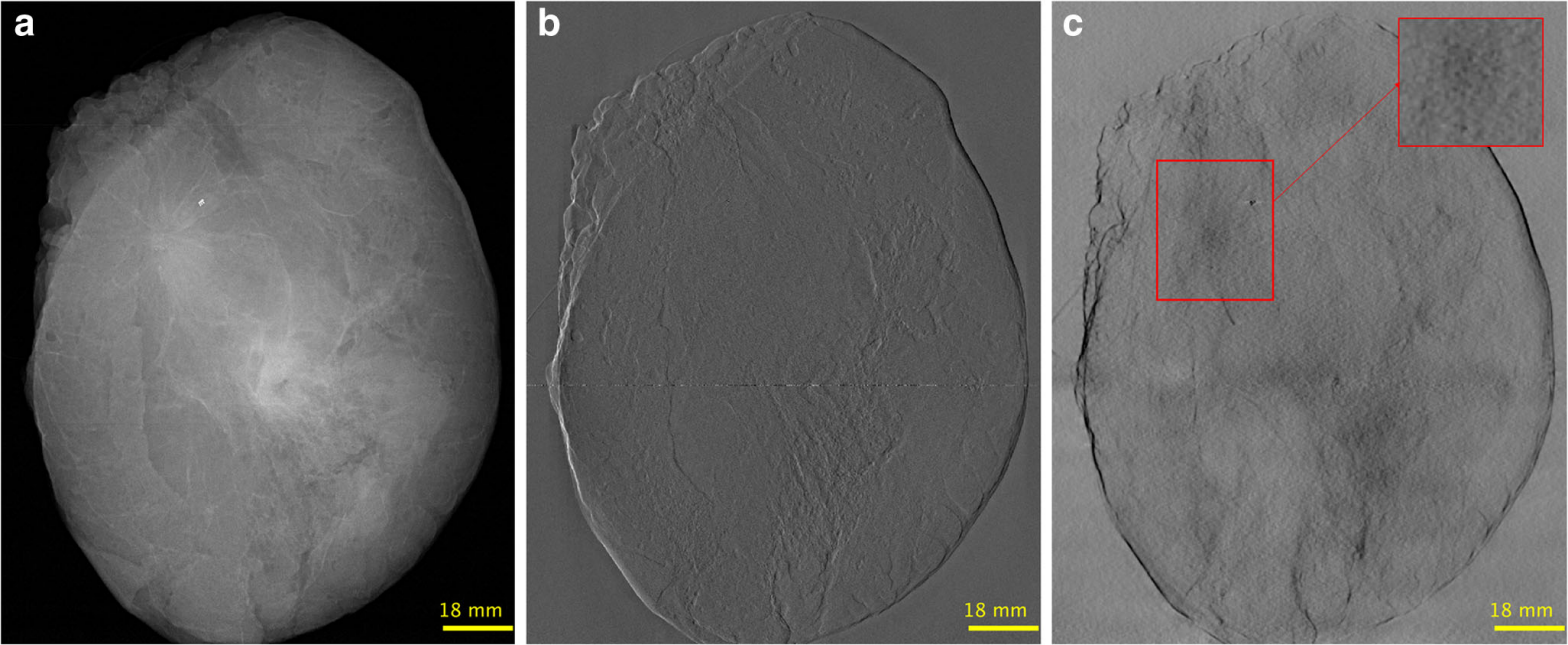
GIM images of sample 2. **a** Attenuation. **b** DPC. **c** DF. The DPC image provides a substantial enhancement of the sample edges, while the DF signal allows a better visualization of the tumor (delimited by the red square, see insert zooming)

**Fig. 4:**
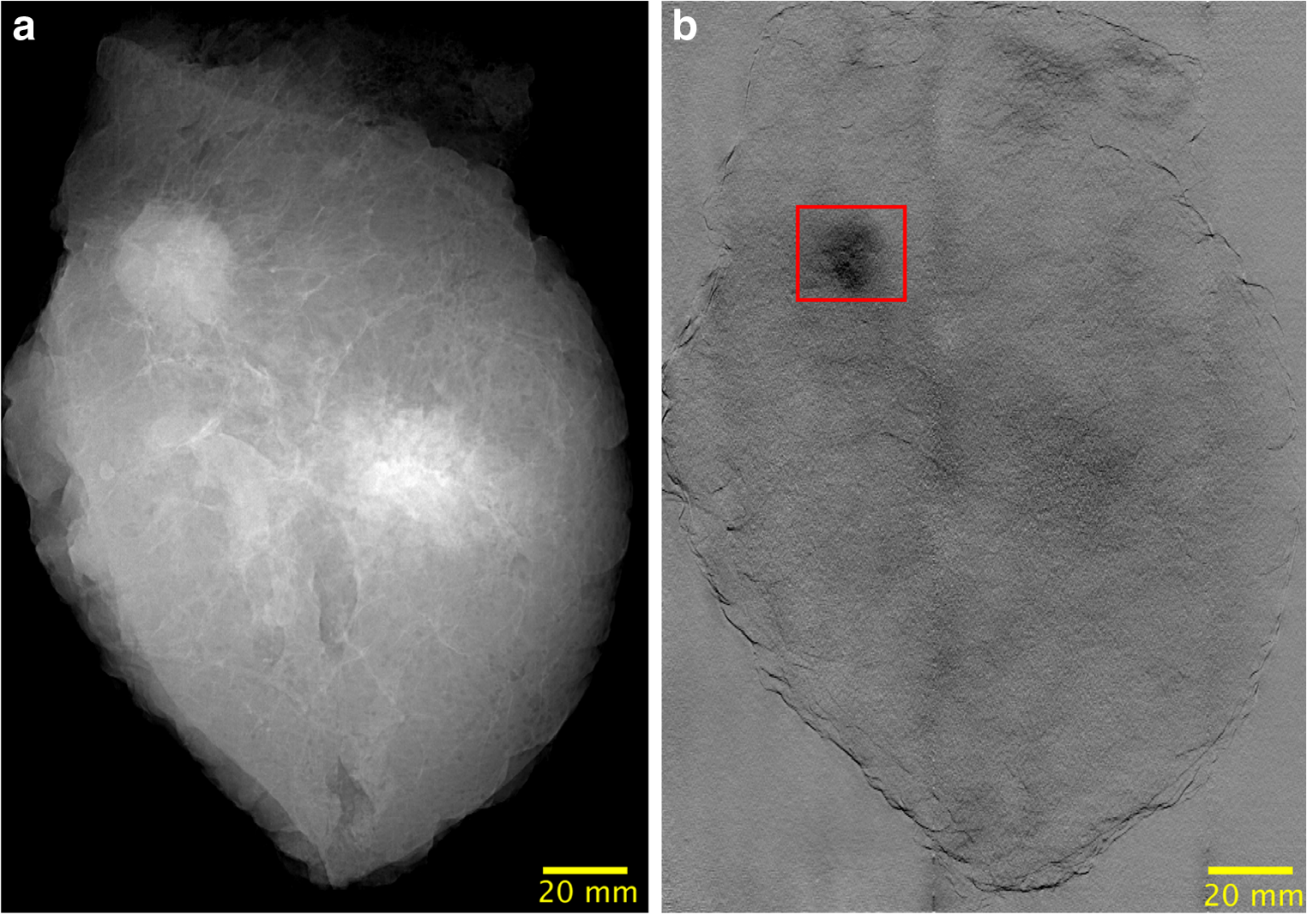
GIM images of sample 4. **a** Attenuation+DPC fused image. **b** DF. The DF image provides a strong signal in the tumor area delineated by the red square, where even calcifications, which are hardly visualized in the fused image, can be observed

In this work, we successfully retrofitted a GI on a standard mammography device. Figures 3 and 4 show the first images obtained with the device. Figure 3 illustrates the attenuation, DPC, and DF signals as produced by the GI add-on. Figure 4 presents the fusion image (obtained by combining the attenuation and DPC signals according to [20]) and the DF signal from another sample.

As evidenced by Figs. 3 and 4, the additional imaging signals provided with GI can yield complementary information at radiation doses comparable to conventional mammography, which could increase the diagnostic performance of the latter. In Fig. 3, it can be observed that the DPC signal facilitates the delineation of edges; this is due to the fact that this signal enhances high spatial frequency components [20], which could contribute to an increased sensitivity for breast cancer detection. The DF signal carries further complementary information, since it can generate contrast enhancement due to the scattering of both tumor masses and microcalcifications. In Fig. 4, the DF signal of the tumor mass was especially prominent showing a higher signal than the fibro-glandular tissue. This holds true potential in the detection of tumors that are embedded in dense breast tissue. Also, the quantification of the DF signal might also enable a non-invasive discrimination of microcalcifications, which would be of great clinical value [10, 22–24].

The corresponding thicknesses, pathological diagnoses, and estimated MGD values for the measured samples are reported in Table 2. The measured air kerma and half-value layer were 5.9 mGy and 0.5 mm Al, respectively. These values were used to calculate the MGD for each sample assuming an 18% glandular density, since the exact breast density was unknown and this is the highest-occurrence density in the population [25]. The resulting MGD values fully comply with the limits established by the European guidelines for the 5-cm breasts, since they report a maximum acceptable dose of less than 2.5 mGy for a 5.3-cm breast. However, the calculated MGD values are slightly above the maximum for the 4-cm specimens, since the Guidelines report a maximum of less than 2.0 mGy for 4.5-cm breasts [14]. However, it is important to consider that a certain breast density (i.e., 18%) was assumed for this dose calculation. If the samples happened to have a glandular density of 36% instead, the dose would be clinically acceptable for the thinner specimens as well. In addition, although the estimated MGDs are slightly higher than those reported for conventional mammography, the values appear similar to those reported for breast tomosynthesis [26].

**Table 2:** Sample characteristics

Sample	Thickness (cm)	Histological diagnosis	MGD (mGy)
1	5 cm	18-mm and 4-mm multicentric invasive ductal carcinoma + DCIS	1.6
2	5 cm	14-mm and 14-mm multicentric invasive ductal carcinoma + DCIS	1.6
3	4 cm	25-mm invasive ductal carcinoma + DCIS	2.0
4	4 cm	38-mm invasive papillary carcinoma mixed with invasive ductal carcinoma + DCIS	2.0

## Discussion

The obtained results demonstrate the feasibility of a clinical application of GIM. From the engineering point of view, the most challenging part of commissioning a GI on an existing scanning-based mammography device was to develop a solid grating holder that allowed keeping the gratings aligned during operation and protected from the vibrations caused by the movement of the scanning arm. In addition to that, it is important to consider that since G0 and G2 are gold gratings, each of them attenuates half of the incoming photons. This means that to obtain SNR values comparable to a standard mammographic setup, it is necessary to increase the exposure times accordingly. The larger the exposure times can be made, while keeping the dose under the acceptable limit [14], the better the quality of the DF and DPC signals. However, it is important to keep in mind that larger exposure times might cause increased patient discomfort and motion artifacts. These aspects shall be considered for the use of this system in an in vivo setting.

Another critical issue is the GI sensitivity that is proportional to the distance between G1 and G2. The fact that this investigational device was commissioned on an existing clinical setup obliged us to fix a few important parameters (like source-to-detector distance, for instance) which imposed additional constraints to the system design. If a GIM device were to be built from scratch, a different geometry could be selected. The disadvantage of the latter option would be that the time between development and transfer to the clinics would become substantially longer.

The presented DF images still have some artifacts, due to the fact that this signal is the most sensitive to noise and vibrations [19], but should only be seen as providing complementary information to that of the anatomical attenuation/fused images, in the same manner as diffusion-weighted images provide supplemental information to anatomical MRI sequences. Nevertheless, further work on improving the DF image quality, e.g., by optimizing image reconstruction and post-processing methods, will increase the usability of this signal.

The purpose of fusing the attenuation and DPC signals (Fig. 4a) is to present an attenuation-like image with additional refraction information to radiologists, so that they can use their experience and knowledge to assess the innovative signals provided with GIM [9]. Notwithstanding, it is a future work to assess in a large reader study whether it is more useful to present the images combined or separately (Fig. 3).

A careful investigation of the optimal acquisition parameters for each different breast thickness and density will be carried out, since most likely voltages higher than those used in traditional mammography would yield better GIM outcomes [8]. In this study, the same exposure settings were used for all the samples, in spite of them having different thicknesses and densities. However, it is important to remember that the variation in the latter two quantities is less prominent in specimen radiography compared with whole breast imaging. In fact, in this particular study, sample thickness ranged from 4 through 5 cm. The decision to always use the same exposure settings was made based on the results of the optimization procedure carried out in [17], in which it was found that the highest flux delivered by the hosting device would yield the best DF and PC quality. However, it might be potentially possible to obtain higher fluxes at lower voltages, in a way that the attenuation CNR would not be sacrificed for the sake of the two additional signals. Before intending to use the system on patients, more mastectomy samples are to be measured to do an accurate evaluation of the diagnostic contribution of GI.

In conclusion, we show first promising results of the successful implementation of a full-FOV clinically compatible GI based on a standardly available FFDM system that might provide complementary information for breast cancer diagnosis. Our approach presently still operates at a higher dose level compared with other systems available on the market. Therefore, the real challenge will be to demonstrate that cancer detection can be considerably increased at this higher dose. However, it is also important to keep in mind that a proper dose calculation, using the Monte Carlo simulations and the appropriate breast phantom, must be carried out as well.

The investigational device has been successfully commissioned and will be soon used for an upcoming in vivo pilot study.

## Abbreviations

DBT: Digital breast tomosynthesis
DF: Dark-field
DPC: Differential phase-contrast
FOV: Field-of-view
GI: Grating interferometry
GIM: Grating-interferometry-based mammography
MGD: Mean glandular dose
MRI: Magnetic resonance imaging
PC: Phase-contrast
PCI: X-ray phase-contrast imaging
SNR: Signal-to-noise ratio
US: Ultrasonography
