# Correction to: Towards clinical grating-interferometry mammography

**DOI:** 10.1007/s00330-019-06476-2

**Published:** 2020-01-02

**Authors:** Carolina Arboleda, Zhentian Wang, Konstantins Jefimovs, Thomas Koehler, Udo Van Stevendaal, Norbert Kuhn, Bernd David, Sven Prevrhal, Kristina Lång, Serafino Forte, Rahel Antonia Kubik-Huch, Cornelia Leo, Gad Singer, Magda Marcon, Andreas Boss, Ewald Roessl, Marco Stampanoni

**Affiliations:** 1 ETH Zurich, Gloriastrasse 35, 8092 Zurich, Switzerland; 2 Paul Scherrer Institute, Forschungsstrasse 111, 5232 Villigen, Switzerland; 3 Philips Research Hamburg, Röntgenstrasse 24-26, 22335 Hamburg, Germany; 4 HAW Hamburg, Ulmenliet 20, 21033 Hamburg, Germany; 5 Department of Radiology, Kantonsspital Baden, Im Ergel 1, 5404 Baden, Switzerland; 6 Interdisciplinary Breast Center, Kantonsspital Baden, Im Ergel 1, 5404 Baden, Switzerland; 7 Department of Pathology, Kantonsspital Baden, Im Ergel 1, 5404 Baden, Switzerland; 8 Institute for Diagnostic and Interventional Radiology, Universitätspital Zurich, Rämistrasse 100, 8091 Zurich, Switzerland

**Correction to: European Radiology**

**https://doi.org/10.1007/s00330-019-06362-x**

The article *Towards clinical grating-interferometry mammography*, written by Carolina Arboleda, Zhentian Wang, Konstantins Jefimovs, Thomas Koehler, Udo Van Stevendaal, Norbert Kuhn, Bernd David, Sven Prevrhal, Kristina Lång, Serafino Forte, Rahel Antonia Kubik-Huch, Cornelia Leo, Gad Singer, Magda Marcon, Andreas Boss, Ewald Roessl, and Marco Stampanoni, was originally published electronically on the publisher’s internet portal (currently SpringerLink) on 22 August 2019 without open access.

With the author(s)’ decision to opt for Open Choice, the copyright of the article changed on 20 December 2019 to © The Author(s) 2019 and the article is forthwith distributed under the terms of the Creative Commons Attribution 4.0 International License (http://creativecommons.org/licenses/by/4.0/), which permits use, duplication, adaptation, distribution, and reproduction in any medium or format, as long as you give appropriate credit to the original author(s) and the source, provide a link to the Creative Commons license, and indicate if changes were made. The original article has been corrected.

